# Antipsychotic treatments; focus on lurasidone

**DOI:** 10.3389/fphar.2013.00102

**Published:** 2013-08-26

**Authors:** Tomiki Sumiyoshi

**Affiliations:** Neurocognition and Pharmacology Laboratory, Department of Neuropsychiatry, University of Toyama Graduate School of Medicine and Pharmaceutical SciencesToyama, Japan

**Keywords:** antipsychotic drugs, second generation, schizophrenia, effectiveness, side effects, remission

## Abstract

The introduction of atypical antipsychotic drugs (AAPDs), or second-generation antipsychotics, with clozapine as the prototype, has largely changed the clinicians' attitudes toward the treatment of mental illnesses including, but not limited to schizophrenia. Initially, there was optimism that AAPDs would be superior over typical antipsychotic drugs (TAPDs), or first-generation antipsychotic drugs, in terms of efficacy in various phenomenological aspects, including cognitive impairment, and less likelihood of causing adverse events. However, these views have been partly challenged by results from recent meta-analysis studies. Specifically, cardio-metabolic side effects of AAPDs, in spite of a relative paucity of extrapyramidal symptoms, may sometimes limit the use of these agents. Accordingly, attempts have been made to develop newer compounds, e.g., lurasidone, with the aim of increasing efficacy and tolerability. Further investigations are warranted to determine if a larger proportion of patients will be benefitted by treatment with AAPDs compared to TAPDs in terms of remission and recovery.

## Introduction

Antipsychotic drugs have been considered to represent a series of compounds to treat specific symptoms of schizophrenia, i.e., positive (delusions, hallucinations, disorganized thoughts, and etc.) and negative (blunt affect, avolition, social withdrawal, and etc.) symptoms. Conventional, or “typical,” antipsychotic drugs (TAPDs) exert antipsychotic effects at doses that cause extrapyramidal motor side effects due to dopamine (DA)-D_2_ receptor blocking properties. Selective actions on psychotic symptoms, with less chance to cause extrapyramidal side effects (EPS), have become possible with the advent of newer class agents, so-called “atypical antipsychotic drugs (AAPDs)” (Meltzer, 1991a). In addition to positive symptoms of schizophrenia, which antipsychotic drugs were initially expected to ameliorate, there is a recent trend to use AAPDs for other psychiatric diseases, e.g., mood disorders, as discussed below.

The development of antipsychotic drugs has been coupled with more intricate theories on the pathophysiology of schizophrenia (Meltzer, 1991b). For example, hyperactivity of DA neurons projecting to the limbic regions, e.g., nucleus accumbens and amygdala, has been shown to be associated with positive symptoms, while a decrease in DA activity in the prefrontal cortex has been considered to cause negative symptoms (Seeman et al., 2006). On the other hand, phencyclidine (PCP), an antagonist at the N-methyl-D-aspartate (NMDA) type glutamate receptor, has been found to produce schizophrenia-like symptoms. This observation led to the glutamate hypothesis of the disease, which is proposed to be linked to the DA hypothesis (Toru et al., 1994).

This article aims to provide theoretical issues on AAPDs in relation to efficacy for treating psychotic symptoms and cognition, as well as safety and tolerability. Specifically, cognitive benefits of lurasidone, a novel AAPD are a focus of this paper. Based on previous discussions (Oliveira et al., 2009; Melnik et al., 2010; Meltzer, 2013) and updated information on these issues, the author present a hypothesis for future directions of therapeutics of schizophrenia and related disorders.

## History of antipsychotic drugs

**Figure 1:**
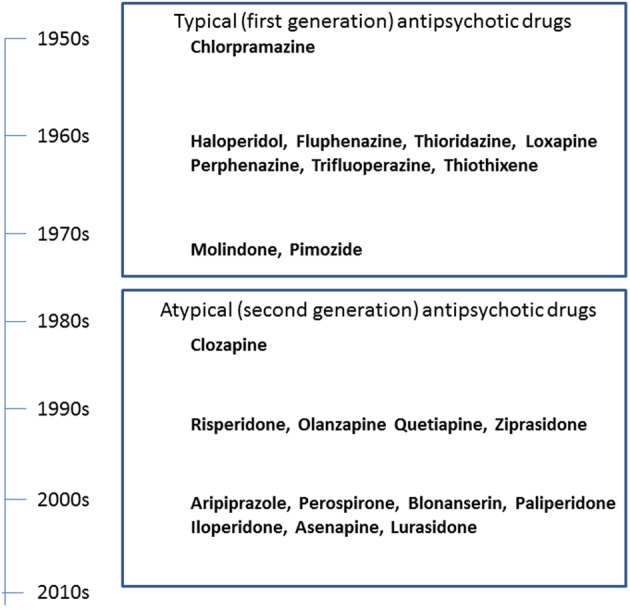
**Historical overview of the development of antipsychotic drugs**.

**Figure 2:**
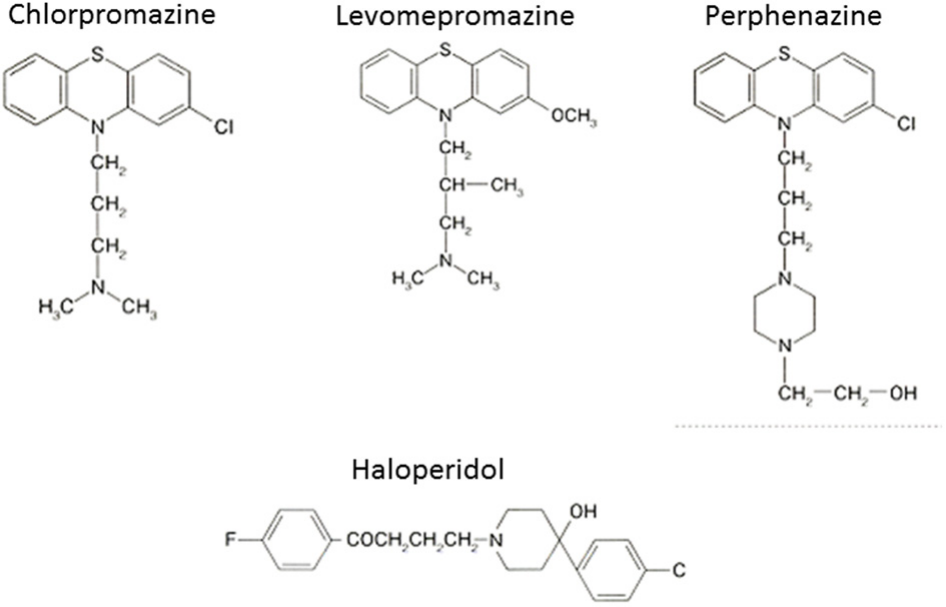
**Representative typical (first generation) antipsychotic drugs**.

The serendipitous discovery of the ability of chlorpromazine to treat psychomotor excitation of schizophrenia confirmed the concept that the illness is a medical entity related to brain chemistry (Delay and Deniker, 1955) (Figure 1). The subsequent development of haloperidol, also inhibiting psychomotor symptoms, provided a clue to the pharmacological target shared by most antipsychotic agents; the DA-D_2_ receptor (Seeman et al., 2006). This property of TAPDs (Figure 2) is associated with the incidence of motor dysfunction, e.g., parkinsonisms, akashisia, dystonia and dyskinesia, as well as endocrinological derangements, e.g., hyperprolactinemia (Sumiyoshi, 2008).

The search for improved medications for schizophrenia led to the implementation of clozapine, the prototype of AAPDs (Kane et al., 1988; Meltzer, 1989). Clozapine shows strong blocking effects for serotonin (5-HT)-5-HT_2A_ and DA-D_4_ receptors relative to D_2_ receptors, which is thought to underlie the ability of this compound to ameliorate not only positive symptoms, but also negative symptoms to some extent, without causing EPS (Meltzer et al., 1989; Stockmeier et al., 1993; Sumiyoshi et al., 1995).

**Figure 3:**
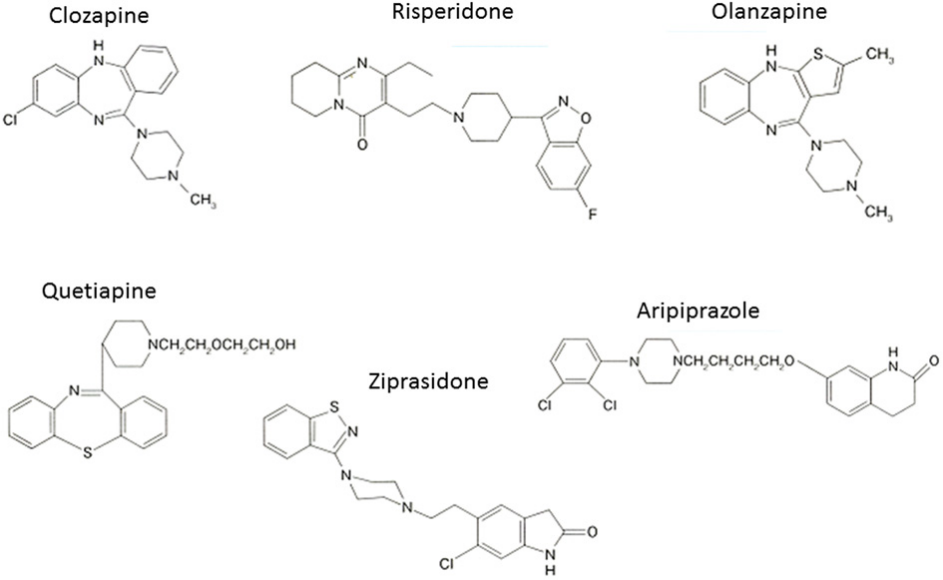
**Atypical (second generation) antipsychotic drugs widely used**.

**Figure 4:**
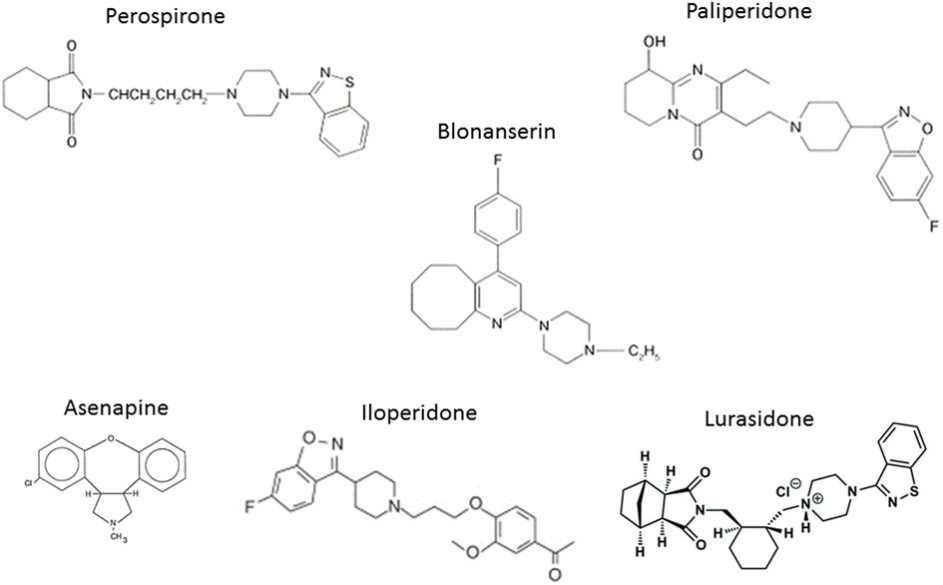
**Atypical (second generation) antipsychotic drugs recently developed**.

The experience with clozapine prompted the development of a series of AAPDs with relatively potent 5-HT_2A_ vs. D_2_ receptor blocking effects, in an attempt to decrease the likelihood of EPS and elevation of plasma prolactin (pPRL) levels. Consequently, risperidone, olanzapine, quetiapine, aripiprazole, ziprasidone, have been developed (Figure 3). In addition, paliperidone, an active metabolite of risperidone, as well as lurasidone, asenapine and iloperidone (in the USA), amisulpiride (in Europe), and perospirone and blonanserin (in Japan), have enriched the choice of AAPDs (Figure 4).

## Pharmacology

**Figure 5:**
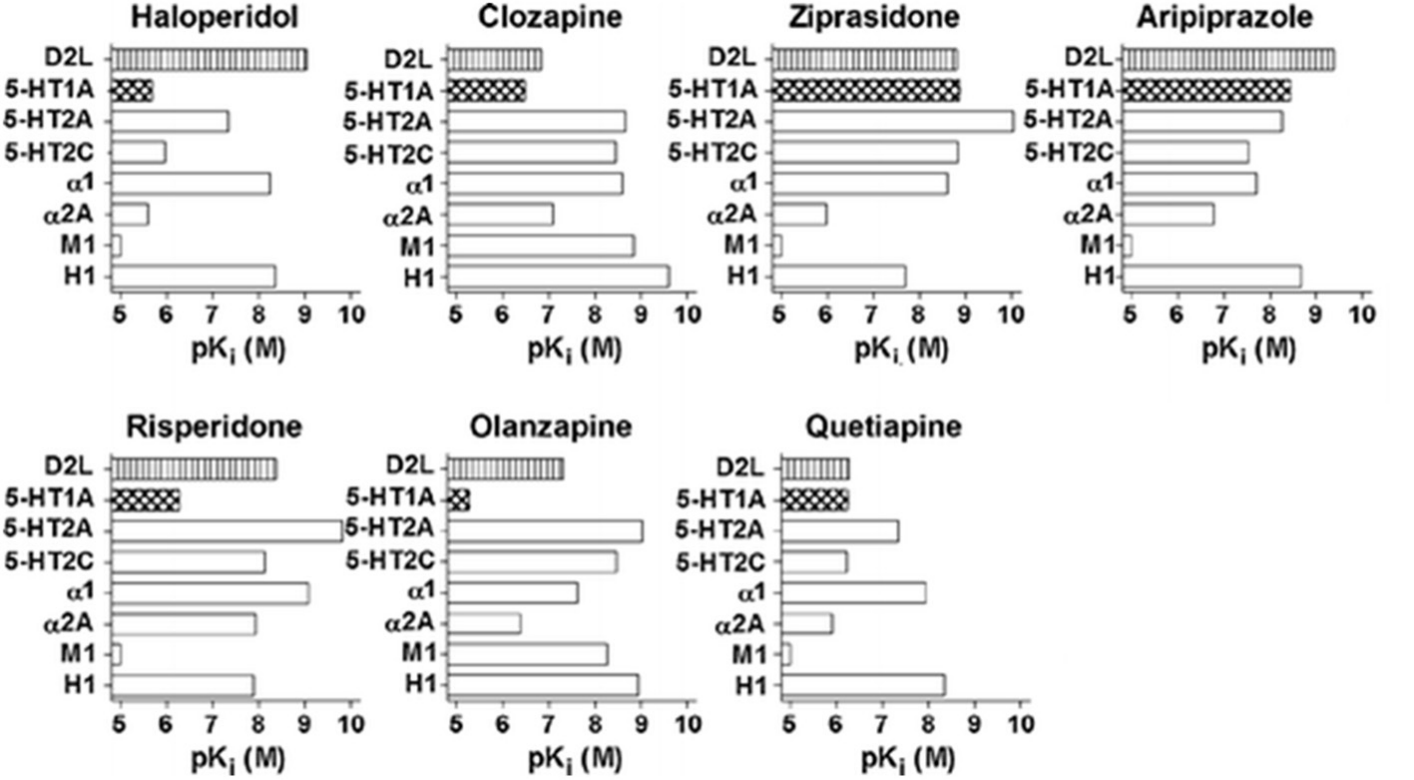
**Receptor binding profiles of antipsychotic drugs.** A larger pKi value represents a stronger affinity for the particular receptor. [Data from Newman-Tancredi and Kleven (2011)].

The above AAPDs, except amisulpiride, a relatively selective D_2_/D_3_ ligand, share a property of relatively high 5-HT_2A_ vs. D_2_ receptor affinity (Meltzer et al., 1989; Stockmeier et al., 1993; Sumiyoshi et al., 1995). Some of them, e.g., clozapine, olanzapine and quetiapine, also exhibit considerable affinities for D_1_, histamine H_1_, adrenalin-α_1_, and muscarinic-M_1_, receptors, and etc. (Meltzer et al., 2003; Newman-Tancredi and Kleven, 2011). Pharmacologic profiles for representative AAPDs can be summarized as eliciting relatively strong affinities for 5-HT_1A_, 5-HT_2C_ and NA-α_1_ receptors, in addition to 5-HT_2A_ and D_2_ receptors, as indicated in Figure 5 (Newman-Tancredi and Kleven, 2011).

Other common pharmacologic features of AAPDs include the ability to increase extracellular concentrations of DA and acetylcholine in the prefrontal cortex, as measured by *in vivo* microdialysis (Kuroki et al., 1998; Ichikawa et al., 2002). This property has been associated with beneficial effects of these compounds on negative symptoms and cognitive impairment (Kuroki et al., 1998; Ichikawa et al., 2002; Meltzer et al., 2003). It should be noted that the mechanisms of action of antipsychotic drugs were largely derived from studies using animal models of behavioral abnormalities, e.g., sensorimotor gating deficits (Swerdlow et al., 1994).

## Efficacy

### General views

A recent meta-analysis comparing AAPDs and TAPDs in the treatment of chronic schizophrenia suggests the advantage of clozapine, risperidone, olanzapine, and amisulpiride over TAPDs (Leucht et al., 2009b) for overall efficacy. However, the effect sizes were small (Leucht et al., 2009b), and specific side effects of these agents, e.g., hyperprolactinemia for risperidone and weight gain/metabolic syndrome for olanzapine and clozapine, should be considered (Zhang et al., 2013).

For first-episode patients, Zhang et al. (2013) conducted a meta-analysis of acute, randomized trials with AAPDs vs. TAPDs comparison. The results indicate AAPDs as a whole showed superior efficacy for negative symptoms, and that olanzapine and amisulpiride specifically showed greater benefits than TAPDs (Zhang et al., 2013).

### Cognition

**Figure 6:**
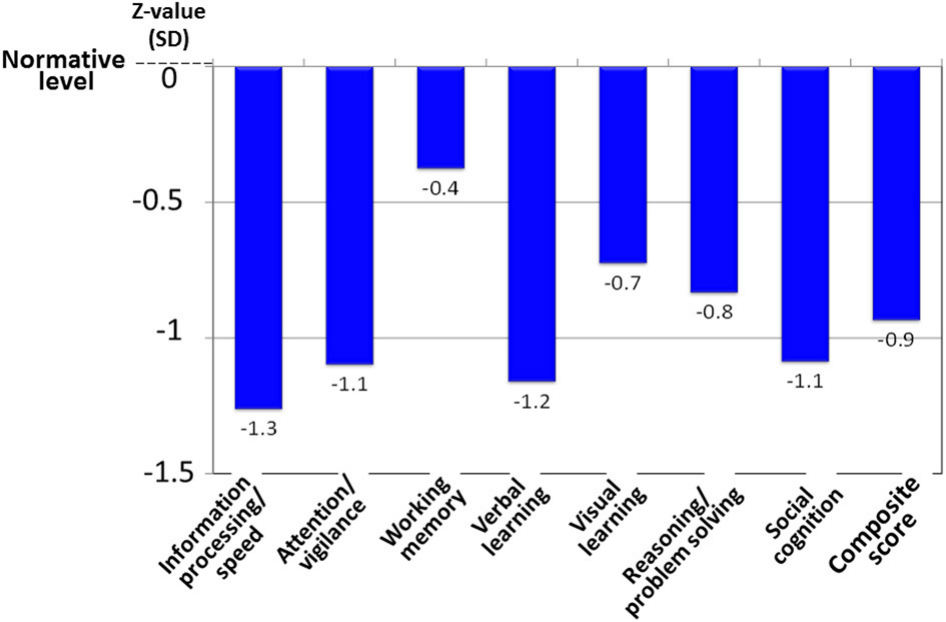
**Cognitive impairment of schizophrenia, as evaluated by the MATRICS Consensus Cognitive Battery–Japanese version.** Patients with schizophrenia (*N* = 30) demonstrate about a one-standard deviation decline from the normative value in the performance on tests of several cognitive domains. Data were obtained from Outpatient Clinic of University of Toyama Hospital.

Patients with schizophrenia demonstrate a 1–2.5 standard deviation decline in performance on neuropsychological tests of a range of cognitive domains, e.g., several types of memory, executive function (planning, flexibility of thinking and etc.), attention/information processing, verbal fluency, and motor function (Harvey and Keefe, 1997; Keefe et al., 2004) (Figure 6). Cognitive impairment in schizophrenia has been suggested to largely determine the outcome for patients (Green, 1996; Addington and Addington, 2000; Green et al., 2000).

Although TAPDs, e.g., haloperidol, exert detrimental influence on cognition in healthy subjects (Saeedi et al., 2006; Veselinovic et al., 2013), there has been controversy about whether AAPDs are more advantageous over TAPDs for its enhancement in schizophrenia (Meltzer et al., 1999; Woodward et al., 2005; Goldberg et al., 2007). Results of the large scale trials, such as the Clinical Antipsychotic Trials of Intervention Effectiveness (CATIE) study, suggest AAPDs may not elicit superiority over TAPDs on cognition (Keefe et al., 2007). However, observations in the CATIE trial should be interpreted with caution, as it did not include a placebo arm, and the results were from chronic patients (Lieberman et al., 2005).

**Figure 7:**
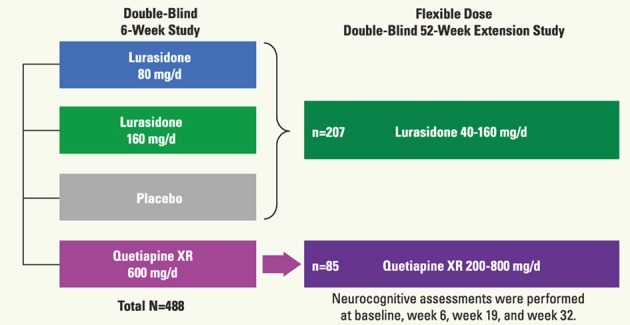
**Schema of the protocol for the double-blind study of the effect of lurasidone on cognitive function in patients with acute schizophrenia (Sumiyoshi et al., 2013; 3rd Asian Congress on Schizophrenia Research)**.

Besides a trial with chronic schizophrenia (Weickert et al., 2003), there has been little study on cognition in acute schizophrenia that includes a placebo arm. Accordingly, we recently reported a double-blind placebo-controlled trial to examine the effect of lurasidone, a novel AAPD (Meyer et al., 2009; Sumiyoshi et al., 2013), on cognitive performance in patients with acute psychosis, followed by a long-term extension study (Sumiyoshi et al., 2013) (Figure 7).

**Figure 8:**
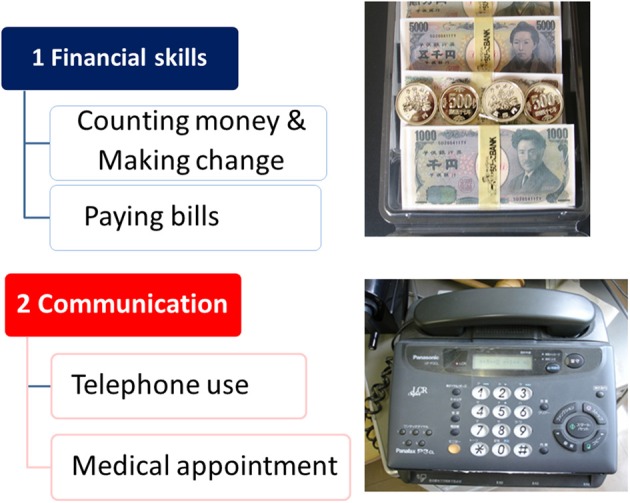
**UCSD Performance-based Skills Assessment—Brief version (UPSA-B)**.

In the acute study patients were randomized to receive treatment with lurasidone 80 mg (*N* = 125), 160 mg (*N* = 121), quetiapine 600 mg (*N* = 120), or placebo (*N* = 122). Subjects who completed the 6-week treatment were eligible for the double-blind extension study to receive a once-daily flexible dose of lurasidone (40–160 mg/day; *N* = 151) or quetiapine (200–800 mg/day; *N* = 85). Subjects who received placebo in the acute study were administered lurasidone (40–160 mg/day; *N* = 56). Cognitive performance was examined with the computerized CogState battery (Pietrzak et al., 2009) at baseline of the acute phase, and after 6, 19, and 32 weeks of treatment. The battery consists of eight tasks that measure verbal learning, speed of processing, attention/vigilance, visual working memory, visual memory, spatial working memory, reasoning and problem solving, and social cognition (Pietrzak et al., 2009). The average of standardized *Z*-scores from each task was used as the valid neurocognitive composite *Z*-score. Functional capacity was evaluated with UCSD Performance-based Skills Assessment—Brief version (UPSA-B) (Mausbach et al., 2011) (Figure 8).

**Figure 9:**
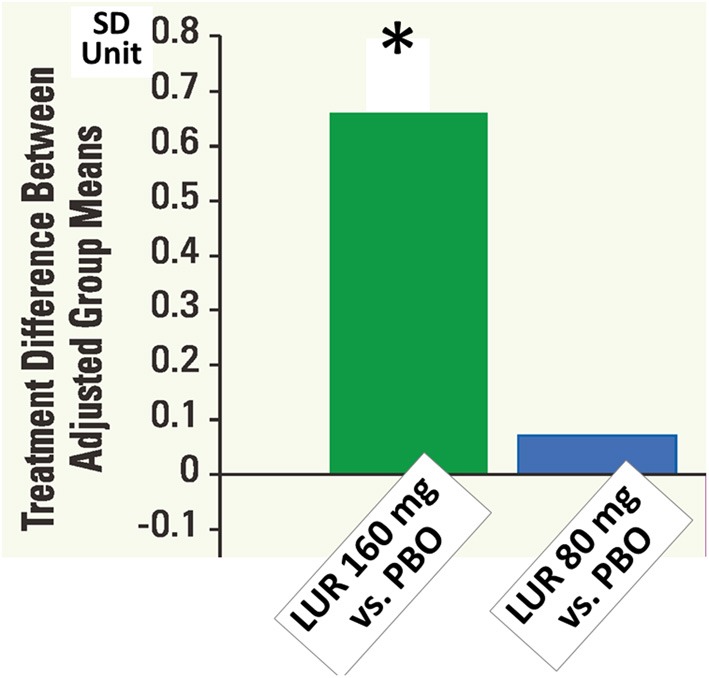
**Cognitive composite z-scores in double-blind 6-week acute phase study of lurasidone (LUR) relative to placebo (PBO).** Data are based on LOCF analysis of CogState composite score (standardized z-score) at week 6 in the evaluable-test sample set (*N* = 267). ^*^P < 0.05, ANCOVA adjusted for baseline and pooled center.

At 6 weeks, the change in the neurocognitive composite *Z-score* did not differ significantly among all groups in intent-to-treat population (*N* = 488). In the evaluable analysis sample (*N* = 267) according to pre-specific criteria, lurasidone, at 160 mg, was superior to both placebo (*p* < 0.05, *d* = 0.367) and quetiapine XR (*p* < 0.05, *d* = 0.411) (Figure 9). Patients with any of the active treatments elicited greater improvement in the UPSA-B score than did those given placebo. In the 6-month extension study, lurasidone, at flexible doses of 40–160 mg/day, showed a significantly greater cognitive benefit compared to quetiapine XR, at flexible doses of 200–800 mg/day, at week 32 (*p* < 0.01, *d* = 0.57). Mixed effects model analysis demonstrated significant cross-sectional and longitudinal relationship between the cognitive composite score and UPSA-B total score.

Data from the placebo-controlled acute phase study provide robust evidence for the ability of lurasidone to enhance cognitive function and functional capacity in patients with schizophrenia. The relatively high rate of subjects who did not provide evaluable data may be associated with awareness of illness, or insight, of study participants (Harvey et al., 2013).

In spite of some beneficial effects, discussed above, no treatments have been approved for treating cognitive or negative symptoms in schizophrenia. Therefore, further efforts are required in this area.

### Mood disorders

Recently, AAPDs have been used for a variety of psychiatric conditions, in addition to schizophrenia, e.g., mood disorders, although the mechanisms underlying their therapeutic effects remain unknown. So far, the Food and Drug Administration in the US has approved indications for olanzapine, quetiapine, risperidone, aripiprazole, and asenapine to treat bipolar disorder, as shown in Table 1 (Bobo, 2013; Spielmans et al., 2013). As for major depressive disorder, a recent meta-analysis (Spielmans et al., 2013) indicates adjunctive treatment with AAPDs, e.g., aripiprazole, olanzapine/fluoxetine, quetiapine, or risperidone, is effective in reducing depressive symptoms, with small-to-moderate effect sizes. Olanzapine, quetiapine, and aripiprazole are indicated to treat major depression (Spielmans et al., 2013), as shown in Table 2.

**Table 1:** **Year of approval by FDA of AAPDs for bipolar disorder**.

	**Acute mania/mixed episodes**	**Bipolar disorder maintenance Tx**	**Acute bipolar depression**
Olanzapine	2000	2004	2003^a^
Quetiapine	2004	2004^b^	2008
Risperidone	2003	2009^c^	
Aripiprazole	2004	2004	
Asenapine	2007		
Lurasidone			2013^d^

a *Olanzapine/flluoxetine combination*.

b *In combination with valproate/lithium*.

c *Depot formulation*.

d *Both for monotherapy and in combination with valproate/lithium*.

**Table 2:** **Year of approval for major depression**.

	**Add-on to antidepressants**	**Monotherapy**
Quetiapine	2009	Applying
Olanzapine	2009^a^	
Aripiprazole	2007	

a *Olanzapine/flluoxetine combination*.

### Other diseases

Some AAPDs have been suggested to ameliorate part of symptoms or caregiver's burden in other conditions, such as Alzheimer's disease (Mohamed et al., 2012), Huntington disease (Adam and Jankovic, 2008), Parkinson's disease (Friedman, 2011), and Tourette's syndrome (Maher and Theodore, 2012). For example, AAPDs have been reported to reduce psychosis, agitation, and/or aggressive behavior in Alzheimer's disease and Huntington disease (Mohamed et al., 2012; Adam and Jankovic, 2008). Clozapine, as well as quetiapine to some extent, has been shown to be effective in controlling psychotic symptoms of Parkinson's disease (Friedman, 2011).

## Tolerability

Compared to TAPDs, AAPDs have been associated with reduced risk of EPS and tardive dyskinesia, although the latter compounds may more frequently induce weight gain and cardio-metabolic side effects in schizophrenia (De Hert et al., 2012a). Further, some large scale studies with chronic patients did not find noticeable differences in efficacy between the two antipsychotic classes (Lieberman et al., 2005; Jones et al., 2006; Leucht et al., 2009a), raising a question about the advantage of AAPDs. However, there is a suggestion that higher benefit/risk ratios for AAPDs would be expected in acute patients compared with chronic patients (Zhang et al., 2013). In fact, a recent meta-analysis (Zhang et al., 2013) indicates olanzapine, amisulpiride, risperidone and quetiapine, elicit superior efficacy, greater treatment persistence and less EPS than TAPDs. These authors also found greater weight increase and metabolic changes for some of these AAPDs, such as olanzapine (Zhang et al., 2013).

These lines of evidence prompted the development of newer antipsychotic drugs with minimal adverse events associated with the above AAPDs, e.g., weight gain, lipid metabolism, cardiovascular risk, and glucose intolerance. Accordingly, the FDA approved iloperidone and asenapine in 2009, followed by lurasidone in 2010, for the treatment of adults with acute schizophrenia. De Hert et al. (2012b) conducted a systematic review and exploratory meta-analysis of these new AAPDs together with paliperidone in the treatment of schizophrenia and bipolar disorder. The findings suggest a relatively greater tolerability for lurasidone in comparison with placebo, and indicate the need for further controlled studies comparing the newer agents with other antipsychotic drugs currently available (De Hert et al., 2012b).

## Perspectives

In the pursuit of novel therapeutics, critical issues to be addressed, or “unmet needs,” include (1) treatment-resistant patients, (2) prevention of psychosis, and (3) remission/recovery. There have been some suggestions for the former two, e.g., clozapine for treatment-resistant schizophrenia (Kane et al., 1988; Meltzer, 1989), and risperidone and olanzapine for prevention (McGorry et al., 2002; McGlashan et al., 2006). On the other hand, there seems to be a relative paucity of information on whether AAPDs increase remission in schizophrenia (Takeuchi et al., 2012), due, partly, to the limited number of valid assessment methods (Alaqeel and Margolese, 2012).

Such measures include the Remission in Schizophrenia Working Group (RSWG) criteria (Andreasen et al., 2005), which has been developed to operationally define symptomatic remission. Using the RSWG criteria, Alaqeel et al. (2013) recently conducted a meta-analysis to compare remission rates between AAPD and TAPD treatments. Results from four eligible studies, with 3433 schizophrenia patients, suggest AAPDs are associated with a 1.46 increased probability of attaining remission relative to TAPDs (Alaqeel et al., 2013). The lower dropout rate with AAPDs may explain the modest but significant increase in the rate of enduring symptomatic remission, which deserves further study.

## Conclusions

**Figure 10:**
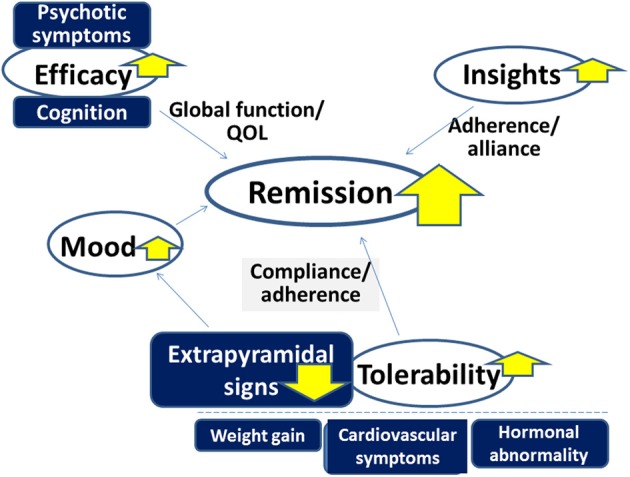
**Advantage of atypical antipsychotic drugs (AAPDs) for improving outcome in the treatment of psychotic disorders.** Compared to typical antipsychotic drugs, AAPDs elicit fewer incidences of extrapyramidal signs, which enhance compliance and adherence. AAPDs may also demonstrate greater efficacy for mood symptoms. The superiority of AAPDs in terms of ameliorating psychotic symptoms and cognitive impairment is under debate. The development of newer antipsychotic drugs with minimal adverse events associated with some existing AAPDs, e.g., weight gain, cardiovascular symptoms, and hormonal abnormalities, may provide an effective strategy to attain greater remission rates.

Antipsychotic drugs play a major role in the treatment of schizophrenia and related disorders. However, there remain a number of issues to be solved to more effectively improve clinical practice, e.g., dealing with treatment-resistant patients. As discussed, some evidence suggests the superiority of AAPDs as a group over TAPDs in terms of compliance/adherence, although controversy exists. At least, it is legitimate to confirm that AAPDs elicit lower incidence of EPS compared to TAPDs. Accordingly, AAPDs may also demonstrate greater efficacy for mood symptoms, and less likelihood to cause secondary negative symptoms related to EPS (Figure 10).

Further investigations are warranted to determine if a larger proportion of patients can be benefitted by treatment with AAPDs compared with TAPDs in terms of remission and recovery. Specifically, efforts to develop newer antipsychotic compounds with minimal adverse events associated with some existing AAPDs, e.g., weight gain, cardiovascular symptoms, and hormonal abnormalities, will provide a promising strategy to attain this goal.

### Conflict of interest statement

The author has received advisory board and/or speaker's honoraria from Dainippon-Sumitomo, Yoshitomi-yakuhin, Tanabe-Mitsubishi, Otsuka, Eli Lilly, and Takeda Pharmaceuticals.
